# Editorial: Advanced Neuroimaging Methods in Brain Disorders

**DOI:** 10.3389/fnhum.2022.906323

**Published:** 2022-04-19

**Authors:** Jurong Ding, Wei Liao, Dajiang Zhu

**Affiliations:** ^1^School of Automation and Information Engineering, Sichuan University of Science and Engineering, Zigong, China; ^2^Artificial Intelligence Key Laboratory of Sichuan Province, Sichuan University of Science and Engineering, Zigong, China; ^3^The Clinical Hospital of Chengdu Brain Science Institute, School of Life Science and Technology, University of Electronic Science and Technology of China, Chengdu, China; ^4^MOE Key Lab for Neuroinformation, High-Field Magnetic Resonance Brain Imaging Key Laboratory of Sichuan Province, University of Electronic Science and Technology of China, Chengdu, China; ^5^Computer Science and Engineering, University of Texas at Arlington, Arlington, TX, United States

**Keywords:** neuroimaging, functional magnetic resonance imaging, brain network, machine learning, biomarkers

The human brain is a large, interactive, and complex system, supporting our daily behavior and cognitive function. Researchers have been working on the mechanisms of the human brain, under healthy conditions and afflicted by different disorders for decades. Advanced neuroimaging techniques, such as magnetoencephalography (MEG), event-related potentials (ERPs), functional near-infrared spectroscopy (fNIRS), and magnetic resonance imaging (MRI), have been extensively utilized to explore the underlying principles of brain structural and functional architectures, as well as pathology alterations in various brain disorders. Especially, multi-modal neuroimaging techniques will in future provide a more comprehensive understanding of pathomechanism of brain disorders than single-modal techniques, which can be useful for early diagnosis and assessment of therapeutic effect and prognosis. In general, the development of robust neuroimaging analytical methods will fundamentally advance scientific understanding of the brain and facilitate numerous neuroscience and clinical research studies.

This Research Topic focuses on the recent developments of neuroimaging methods and their applications in various domains. In total, 19 contributions, including 16 original research articles, 2 study protocols, and one correction, were collected from internationally recognized researchers in the field.

## Magnetoencephalography (MEG) and Event-Related Potentials (ERPs)

Using MEG recordings, Niu et al. explored the impact of antiepileptic drugs on cognition and neuromagnetic activities in children suffering from childhood epilepsy with centrotemporal spikes (CECTS). In this longitudinal study, children with CECTS after 1 year of treatment showed increased scores on cognitive assessment, as well as enhanced neural activity and functional connectivity of the default mode network (DMN). These results demonstrated that the normalization of aberrant DMN caused by antiepileptic drugs in children with CECTS likely explains the improvement of cognitive function.

Event-related potential (ERPs) reflect brain activity and can be used to explore cognitive function. By using the ERPs technique, Wen et al. investigated the cognitive function and psychosocial factors between patients with major depressive disorder (MDD) with/without non-suicidal self-injury (NSSI) behavior and healthy controls. They found that patients with MDD with NSSI behavior exhibited impaired cognitive function, such as memory decline, attention and executive function deficits, and low-interference ability. The psychosocial factors contributing to NSSI behavior in adolescents with MDD were childhood abuse, lack of social support, and a sense of shame. Their findings highlight the risk factors for MDD with NSSI behavior, which can help the diagnosis and treatment of these patients.

## Functional Near-Infrared Spectroscopy (fNIRS)

fNIRS, an optical neuroimaging technique, can provide quantitative hemodynamic responses to map the functional state of the brain. Zhang et al. proposed a quantitative power analysis model combining fNIRS technique and the Welch power spectrum estimation method to examine the brain functional activation of deceptive behavior. They found that spontaneous deceptive behaviors exhibited significantly higher power than controlled behavior. Meanwhile, the power existed a significant difference between the three cases (win-win, lose-win, and lose-lose conditions) for the spontaneous deceptive behavior not for the controlled behavior, suggesting that the reward system was only involved in the deception.

Using the fNIRS technique, Sun et al. evaluated narrowband resting-state functional connectivity (RSFC) at 14 frequency bands in 25 children with autism spectrum disorder (ASD) and 22 typically developing (TD) children. They reported that interhemispheric RSFC in several frequency bands was significantly decreased in ASD children. The difference in RSFC between the two groups was more pronounced in 0.01–0.02 Hz than in the other frequency bands, and the receiver operating characteristic analysis further demonstrated that 0.01–0.02 Hz RSFC showed an excellent discrimination ability between ASD and TD children, indicating that the narrowband RSFC might be a potential predictor of ASD.

## Magnetic Resonance Imaging (MRI)

Several MRI techniques, including magnetic resonance spectroscopy (MRS), susceptibility-weighted imaging (SWI), structural MRI, functional MRI (fMRI), and diffusion tensor imaging (DTI), can capture metabolic, functional, and structural changes in the brain and have caught the widespread attention of researchers. Using MRS, Sharma et al. investigated intra-subject reproducibility and repeatability of brain temperature. Their findings prove that MRS is a reliable and reproducible approach to measuring brain temperature, and may be utilized to non-invasively monitor brain temperature changes in health and disease. SWI is a useful tool for estimating iron deposition in the brain. One study used the SWI technique and machine learning methods to explore the relationship of brain iron deposition with the progression of Alzheimer's disease (AD) and found that AD progression-related regions were consistent with the regions reported from previous genetic association studies. Moreover, this study reported a new potential AD-related gene (MEF2C) (You et al.).

Using volumetric and vertex-wise shape analysis of structural MRI data, Gu et al. reported that patients with end-stage renal disease have subcortical structural atrophy, and the atrophy of the thalamus and accumbens was associated with cognitive dysfunction and sleep disorders, respectively. A structural covariance network (SCN) based on structural MRI data can characterize the synchronized topological patterns of brain regions during healthy or diseased states. To facilitate the use of SCN, Xu et al. developed a user-friendly graphical user interface software named “Brain Covariance Connectivity Toolkit” (BCCT) based on the MATLAB platform, which contained the SCN analysis, group comparison, and result-showing modules. This toolkit is convenient for researchers, especially clinical researchers, to perform brain covariance connectivity analysis.

Three resting-state fMRI (rs-fMRI) studies examined brain dysfunction in patients with different diseases. By measuring the static and dynamic amplitude of low-frequency fluctuations of rs-fMRI data in patients with chronic obstructive pulmonary disease, Lv et al. observed the altered static and dynamic brain activities in the basal ganglia and parahippocampal/hippocampal cortex in patients, as well as the relationship of these alterations with poor pulmonary function and semantic-memory impairments. Dry eye disease (DED) was investigated by combining rs-fMRI analysis methods and support vector machines in two studies. Yan et al. reported that patients with DED exhibited abnormal regional homogeneity (ReHo) values in the limbic-cortical circuits and the combination of ReHo values in the left middle cingulum and the left inferior occipital gyrus could be a neuroimaging biomarker to distinguish patients from healthy controls. In the study of Pan et al., reduced global-brain functional connectivity (FC) of the cerebello-thalamo-cortical network was found in patients with DED, and the FC values in the left thalamus can discriminate patients from controls with optimal accuracy, sensitivity, and specificity.

## Multi-Modal Neuroimaging Techniques

Compared to single-modal measures, multi-modal neuroimaging analysis can provide complementary information to uncover the underlying brain mechanism. Qian et al. combined voxel-based morphometry (VBM) analysis and whole-volume psycho-physiological interactions analysis in multi-modal MRI data to assess gray matter volumes and FC alterations in individuals with betel quid dependency (BQD). Their results demonstrated that the caudate could be the structural basis of trait impulsivity and there existed abnormal frontostriatal connectivity in individuals with BQD. Combining structural and rs-fMRI techniques, Li et al. reported common and specific functional alterations of the amygdala between MDD patients with and without anxiety, which were dependent on different amygdala subdivisions.

By combining DTI and rs-fMRI techniques, Wang et al. utilized the Dense Individualized and Common Connectivity-Based Cortical Landmarks (DICCCOL) method to evaluate fine-granularity structural and functional alterations in patients with attention deficit hyperactivity disorder (ADHD) from two independent samples. They observed four neighboring DICCCOLs near the left parietooccipital area with consistently discrepant fiber bundles and 78 consistent abnormal functional connectivities in ADHD patients across the two datasets. Their study provides a new approach for a multicenter and large sample ADHD study in the future. Using multi-modal neuroimaging data, Hu et al. examined gray matter volumes, seed-based FC, and DTI metrics (i.e., fractional anisotropy, axial, mean, and radial diffusivities) in patients with Wilson's disease. Their results revealed that aberrant white matter tracks in association fibers may account for prospective memory impairment, and gray matter volume changes in the association cortex have an indirect impact on global cognitive function by its abnormal FC in patients.

Pietrosanu et al. proposed a sparse group lasso method for multiple imaging modalities analysis to increase interpretability, generalizability, and stability and proved the advantage of their approach over independent sparse and joint sparse models in both simulation and neuroimaging results. Their study provides a stable and interpretable multi-modal imaging analysis framework for detecting brain structural changes in disease. In addition, two research teams shared their study protocol. Walker et al. described their study protocol for the Teen Inflammation Glutamate Emotion Research (TIGER) project, which will collect clinical, behavioral, and multi-modal imaging data to evaluate the associations among inflammation, glutamate levels, and neurodevelopmental trajectories in adolescents with depression. In a recent study protocol, Hu et al. reported the procedures for a prospective, multicenter, longitudinal cohort study, which aimed to establish an individual-based model for the prediction of communication impairment in children with bilateral cerebral palsy and periventricular white matter lesions at school entry by using machine learning algorithms and multi-modal MRI data acquired at an early age.

To summarize, the series of research articles and study protocols absorbed by this Research Topic shows the recent exciting achievements of advanced neuroimaging techniques and applications. We hope that these works will enrich our understanding of physiological and pathological mechanisms of the human brain from a neuroimaging perspective, and will further prompt the development of new imaging analytical methods and their application in more disease types.

## Author Contributions

All authors listed have made a substantial, direct, and intellectual contribution to the work and approved it for publication.

## Funding

Grants from the Scientific Research Foundation of Sichuan University of Science and Engineering (2020RC31) supported JD.

## Conflict of Interest

The authors declare that the research was conducted in the absence of any commercial or financial relationships that could be construed as a potential conflict of interest.

## Publisher's Note

All claims expressed in this article are solely those of the authors and do not necessarily represent those of their affiliated organizations, or those of the publisher, the editors and the reviewers. Any product that may be evaluated in this article, or claim that may be made by its manufacturer, is not guaranteed or endorsed by the publisher.

